# Characterization of the Role of a Non-GPCR Membrane-Bound CFEM Protein in the Pathogenicity and Germination of *Botrytis cinerea*

**DOI:** 10.3390/microorganisms8071043

**Published:** 2020-07-14

**Authors:** Gulab Chand Arya, Dhruv Aditya Srivastava, Eswari P. J. Pandaranayaka, Ekaterina Manasherova, Dov Bernard Prusky, Yigal Elad, Omer Frenkel, Hay Dvir, Arye Harel

**Affiliations:** 1 Department of Vegetable and Field Crops, Institute of Plant Sciences, Agricultural Research Organization, Volcani Center, Rishon LeZion 7505101, Israel; garya56@gmail.com (G.C.A.); dhruv.srivastava@mail.huji.ac.il (D.A.S.); eswaripj@gmail.com (E.P.J.P.); ekaterina@volcani.agri.gov.il (E.M.); 2 Department of Postharvest Science, Institute of Postharvest and Food Sciences, Agricultural Research Organization, Volcani Center, Rishon LeZion7505101, Israel; dovprusk@volcani.agri.gov.il; 3 Department of Plant Pathology and Weed Research, Institute of Plant Protection, Agricultural Research Organization, Volcani Center, Rishon LeZion 7505101, Israel; elady@volcani.agri.gov.il (Y.E.); omerf@volcani.agri.gov.il (O.F.); 4 Department of Ruminant Science, Institute of Animal Science, Agricultural Research Organization, Volcani Center, Rishon LeZion 7505101, Israel; haydvir@volcani.agri.gov.il

**Keywords:** *Botrytis cinerea*, virulence, CFEM, germination, pathogenicity

## Abstract

The necrotrophic fungus *Botrytis cinerea*, is considered a major cause of postharvest losses in a wide range of crops. The common fungal extracellular membrane protein (CFEM), containing a conserved eight-cysteine pattern, was found exclusively in fungi. Previous studies in phytopathogenic fungi have demonstrated the role of membrane-bound and secreted CFEM-containing proteins in different aspects of fungal virulence. However, non-G protein-coupled receptor (non-GPCR) membrane CFEM proteins have not been studied yet in phytopathogenic fungi. In the present study, we have identified a non-GPCR membrane-bound CFEM-containing protein, Bcin07g03260, in the *B. cinerea* genome, and generated deletion mutants, ΔCFEM-*Bcin07g03260*, to study its potential role in physiology and virulence. Three independent ΔCFEM-*Bcin07g03260* mutants showed significantly reduced progression of a necrotic lesion on tomato (*Solanum lycopersicum*) leaves. Further analysis of the mutants revealed significant reduction (approximately 20–30%) in conidial germination and consequent germ tube elongation compared with the WT. Our data complements a previous study of secreted Δ*CFEM1* mutants of *B. cinerea* that showed reduced progression of necrotic lesions on leaves, without effect on germination. Considering various functions identified for CFEM proteins in fungal virulence, our work illustrates a potential new role for a non-GPCR membrane CFEM in pathogenic fungi to control virulence in the fungus *B. cinerea*.

## 1. Introduction

*Botrytis cinerea* (teleomorph: *Botryotinia fuckeliana*), the causal agent of gray mold, is a necrotrophic fungal pathogen that is considered a major cause of postharvest losses in a wide range of crops including fruits, vegetables, and flowers [1]. Important factors contributing to its success as a postharvest pathogen are conducive conditions prevailing throughout the handling pipeline, including humidity, injuries, senescing plant tissue and high sugar content. Considerable postharvest losses, due to *B. cinerea* have been found in: blackberry, blueberry, currant, grape, kiwi, pomegranate, quince, raspberries, strawberry, grapes and many other crops [2,3]. Capable of infecting over 580 genera of plants (including agriculturally important crops) [2,4,5], *B. cinerea* causes necrotic lesions in foliage and other plant parts, ultimately leading to plant death [6,7]. Taken together with the importance of this pathogen, and availability of molecular tools [8] and the genome [9], supporting functional analysis, it has become an important model for molecular studies of necrotrophic fungi.

The common fungal extracellular membrane proteins (CFEM), containing a conserved eight-cysteine pattern [10], have been found exclusively in fungi (mainly Ascomycota, and also Basidiomycota) [10,11,12,13], and they were found to be enriched in pathogenic fungi [14]. However, this domain was found to participate in various functions mediating different physiological (e.g., cell wall stability [15,16]) and infection processes [10,17,18,19]. Studies in the mammal pathogens *Candida albicans* and *C. parapsilosis* illustrated that a subclass of the CFEM family of proteins are involved in utilization of heme and hemoglobin as iron sources [11,20,21]. Additional studies suggested that *Candida* CFEM proteins form a relay network of heme-binding proteins for the shuttling of heme across the fungal cell wall for its cellular assimilation [17,22,23]. These CFEM proteins contain a secreted Csa2, glycosylphosphatidylinositol (GPI) cell wall-bound Rbt5, and the more internal cell wall and plasma membrane anchored Pga7.

Studies of phytopathogenic fungi demonstrated the role of membrane-bound and secreted CFEM-containing proteins involved in different aspects of virulence. The membrane-bound proteins studied were the G-protein coupled receptor (GPCR, containing 7 transmembrane domains [24]) CFEM-containing proteins (Pth11 and Pth11-like) in *Magnaporthe oryzae* and *Fusarium graminearum* [18,25,26]. These studies illustrated that GPCR CFEM Pth11 and Pth11-like proteins are required for proper development of the appressoria, and pathogenicity in *M. oryzae* [18,26]. A mutant of the CFEM-containing Pth11-like receptor in *F. graminearum* also showed reduced virulence. In contrast to GPCR CFEM mutants in *M. oryzae*, the mutant of the *F. graminearum* homolog did not show impaired penetration [25]. This difference demonstrates the variability of homologs of CFEM GPCR Pth11, which are able to regulate virulence via different physiological/molecular pathways in different fungi. Other studies of secreted CFEM-containing proteins illustrated their role in *Magnaporthe grisea* and *B. cinerea* [19,27]. The adenylate cyclase (MAC1) CFEM-containing secreted protein was shown to regulate appressorium formation in *M*. *grisea* [27]. Finally, in *B. cinerea*, the transient expression of a gene encoding for the extracellular CFEM protein *CFEM1* induces chlorosis in *Nicotiana benthamiana* leaves (using the *Agrobacterium* infiltration method), and deletion of *CFEM1* reduced the necrotic progression rate in French bean (*Phaseolus vulgaris*) [19].

Studies so far have not included analysis of a non-GPCR membrane CFEM protein (or a CFEM protein with only one transmembrane domain) in phytopathogenic fungi. Considering the various functions of CFEM proteins in fungal virulence, a non-GPCR membrane subclass may have an important role in pathogenicity via an unknown pathway. Thus, the objectives of the current study were to identify a non-GPCR membrane CFEM protein in *B. cinerea*, and to characterize its role in physiology and pathogenicity.

## 2. Material and Methods

### 2.1. Fungal and Plant Material

*Botrytis cinerea* strain B05.10 [28], used throughout this study (from here on referred to as WT), was routinely cultured on Potato dextrose agar (PDA, Difco, BD, Sparks, MD, USA; adjusted to 2% agar; with 0.25 *w*/*v* chloramphenicol, Sigma-Aldrich, St. Louis, MO, USA) at 18 °C in the dark, unless otherwise specified. Tomato (*Solanum lycopersicum,* cv. Money Maker) seedlings were routinely grown in growth chambers at 25 °C for 4–5 weeks (under 16/8 h fluorescent-based light/dark regimes in Green quality soil mix, Tuff soil, Merom Golan, Israel) and supplied once a week with 3 mL/L fertilizer containing 4% nitrogen, 2% phosphorus, 5% potassium, 6% trace elements, magnesium 0.9% and calcium 1.5%. Plants were transplanted to 2-L plastic pots in the walk-in growth chamber controlled at 22 ± 1 °C with 16/8 h of fluorescent-based light/dark regimes, acclimatized for one week before inoculation.

### 2.2. Identification of a Non-GPCR Membrane-Bound CFEM-Containing Protein

We utilized the standalone InterProScan [29] to identify three protein-coding genes containing a membrane-bound CFEM domain (Table 1) in the *B. cinerea* genome [9]. To further validate prediction of membrane association we utilized a WoLF PSORT subcellular localization predictor (using default parameters) [30], and predictions for transmembrane (TM) regions based on TMHMM [31]. Predictions of the association with the cell wall-related glycosylphosphatidylinositol moiety anchored to the encoded protein was generated using PredGPI server [32] using default parameters. Proteins containing a transmembrane domain, which were also predicted to reside in the plasma membrane by WoLF PSORT, and were not predicted to contain a (cell wall-associated) GPI anchor site, were considered as membrane bound proteins (from here on referred to as membrane protein(s); see, final location prediction in Table 1) [33]. The presence of the CFEM conserved pattern of eight cysteines [10] was further validated by multiple sequence alignment (using MAFFT with default parameters, [34]) for the 3 *B. cinerea* membrane proteins containing InterPro CFEM domains (Table 1) [35], compared with the previously annotated *C. albicans CSA2* gene [23,36]. All three proteins contain the conserved aspartic residue essential for ligation activity (see, [17,23]). Since GPCR proteins contain 7 transmembrane domains [24], we decided to exclude Bcin05g02420 and Bcin15g02580 proteins, which are predicted to contain 6 or 8 transmembrane domains, respectively (Table 1). Although they were not predicted as GPCRs, they have higher likelihood of containing 7 transmembrane domains in case of an error in the prediction. Bcin15g02580 for example is predicted to contain only 7 transmembrane domains according to InterPro (but 8 according to TMHMM). Finally, we selected CFEM-Bcin07g03260, comprising a single transmembrane domain for all downstream analysis of this study.

### 2.3. Infection Assay

The adaxial side of tomato plant leaves were inoculated with 1000 conidia of *B. cinerea* (six inoculation sites per leaf, four leaves per plant), suspended in 10 µL of 1/4 PDB (Potato dextrose broth, Difco, BD, Sparks, MD, USA, pH 6.4) [37]. Disease progression was monitored at 0, 16, and 48 h post infection (hpi). Pathogenicity assays of *B. cinerea* on tomato leaves were performed with the conidia harvested from 10 day-old agar plates (PDA). For detached leaf infection assays used to test mutant levels of pathogenicity, we used leaves from 4 week-old tomato plants that were placed in petri dishes containing moist blotting paper and inoculated them with 10 µL conidial suspensions (described above). The lesion area was calculated at different time points using Image J software (NIH).

### 2.4. DNA and RNA Isolation

Fungal mycelium was grown on PDA plates with cellophane paper. Mycelium were scratched from the plates, grounded in liquid N_2_ and used for DNA or RNA isolation. DNA was isolated using DNA lysis buffer at pH 8.0 containing Tris-HCl (200 mM), NaCl (250 mM), EDTA (25 mM), and SDS (0.001% *v*/*v*). DNA was digested with RNAse and used for PCR. RNA was extracted using the Plant/Fungal RNA isolation Kit (cat–25800, Norgen, Canada), following the manufacturer’s instructions, followed by DNase treatment (Qiagen kit, cat–79254, Hilden, Germany).

### 2.5. Quantitative RT-PCR

cDNAs were made using Thermo Scientific Maxima First strand kit (cat–K1671, Baltics, UAB, Lithuania) from 1000 ng total RNA following the manufacturer’s instructions. The qPCR (qRT-PCR) reactions were carried out as follows: 10 min at 94 °C, and 40 cycles of 94 °C for 10 s, 59 °C for 15 s, and 72 °C for 20 s (Rotor-Gene 6000 Corbett Research, Australia) using gene specific primers (Table 2, see more details about each experiment in figure legends). ABsolute Blue qPCR Mix, SYBR Green (Thermo, cat–AB4322B, Baltics, UAB, Lithuania) was used in the qPCR reactions. The ubiquitin-conjugating enzyme gene (UCE) [38] that was used as a reference gene is illustrated in all relevant figures. The UCE gene was selected because it was shown (together with the ubiquitin gene UBQ) to have the best stability of gene expression across various conditions that are relevant to the *B. cinerea* infection process, when compared to other commonly used control genes (i.e., glyceraldehyde-3-phosphate dehydrogenase; β-tubulin; actin, and α-tubulin genes) [38]. The samples were subjected to melting-curve analysis: efficiencies were close to 100% for all selected primer pairs (Table 2). Relative expression was calculated using the −ΔΔ*C*_T_ method.

### 2.6. Construction of CFEM-Bcin07g03260 Mutants

**Figure 1:**
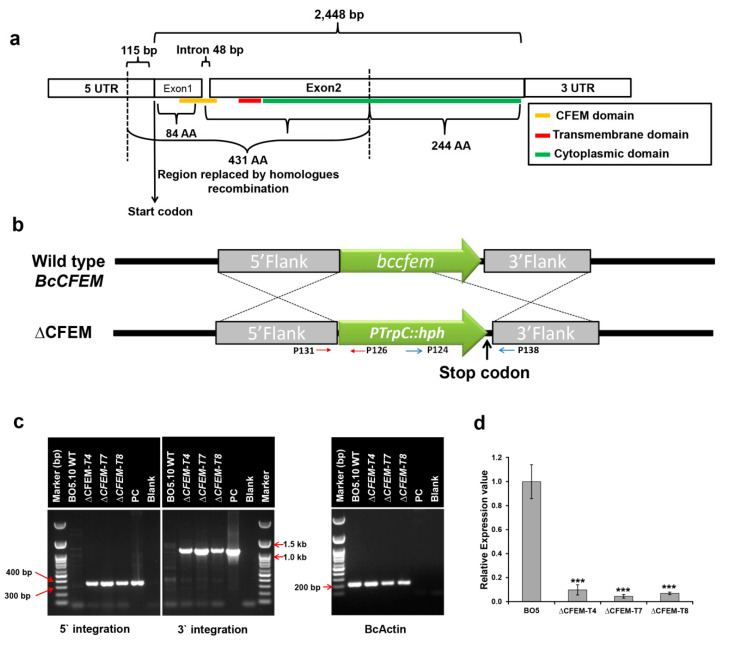
Identification and deletion of the CFEM-*Bcin07g03260* gene in WT *B.* cinerea. (**a**) Genomic arrangement of the CFEM-*Bcin07g03260* gene in the *B. cinerea* genome showing the CFEM domain (yellow), transmembrane domain (red), cytoplasmic domain (green) and the region that was replaced by homologous recombination (1456 bp). (**b**) Homologous recombination-based construct used for deletion of the *Bcin07g03260* gene. The 5′ and 3′ flanks were amplified (with restriction sites) from genomic DNA and cloned in either side of the Hyg cassette, which was used to transform WT *B. cinerea* protoplasts. Red, and blue arrows indicate primers used for 5′ and 3′ integration, respectively. (**c**) Diagnostic PCR showing the successful integration of the Hyg cassette (PtrpC:hph) in the *B. cinerea* genome. The primers used to confirm the integration (Table 2) are indicated in panel B (5′ integration—red arrow, and 3′ integration—blue arrow; PC-Plasmid control for PCR). (**d**) Real-time qPCR showing negligible expression of the CFEM-*Bcin07g03260* gene transcript in the deletion mutants. The transcript level was determined using gene specific primers (Table 2). The expression was normalized using expression of *B. cinerea* UCE. The expression analysis was performed on three biological replicates, each containing three technical repeats. Bars indicate standard error (SE) (*** indicate *p* < 0.001, obtained by Student’s T-test).

For deletion of the CFEM-*Bcin07g03260* gene, we employed a replacement that is based on homologous recombination. Two flanking regions were cloned in either side of the hygromycin resistance cassette (HygR, containing hygromycin phosphotransferase gene, *hph,* under PtrpC promoter) that was obtained from the pNDH-AGT vector [8]: 1043 bp 5′ flank region upstream to −115 bp from start codon (from promoter region to ensure no expression has been driven), and 1000 bp 3′ flank region including the untranslated region (3′ UTR) (see results Figure 1a). The flanking regions were extracted by PCR on *B. cinerea* genomic DNA using primers that contain restriction sites (Table 2) that facilitated ligation into both sides of HygR present inside multiple cloning site (MCS) of the pMA-RQ vector (Invitrogen, USA). The construct was designed to replace the first 431 amino acids (out of 675) of the coding region, which includes the entire CFEM domain, all of exon 1 and 60% of exon 2 of the CFEM*-Bcin07g03260* gene, including its transmembrane domain (Figure 1a). The replacement vector (Figure 1a,b) was transformed into WT *B. cinerea* protoplasts using previously described protocols for protoplast generation and transformation [8]. Single conidium isolates were screened for identification of homokaryon transformants (see [8]). Homologous integration events in hygromycin-resistant transformants were detected by diagnostic PCR using the following primer pairs: P131FP–P126RP to detect 5′ flank integration; and P124FP–P138RP to detect 3′ flank integration (Table 2, Figure 1c). Replacement of the first 115 nucleotides upstream of the coding region (together with the consequent coding region) was likely to impair the transcription initiation process. Ensembl genome browser (https://fungi.ensembl.org/) was used to validate that the 115 nucleotides upstream fragment is not in another gene. The translation process in the deletion mutant would also have been stopped by the Hyg stop codon, which is present in the hygromycin resistance cassette used in this study (as depicted in the revised Figure 1b). The consequent replacement of all of exon 1 and 60% of exon 2 (together encoding for 431 amino acids) of the CFEM-Bcin07g03260 also ensured the removal of all of the CFEM domain (spanning exons 1 and 2). Therefore, even if the remaining part of the gene that was not deleted (encoding for 244 amino acids in exon 2) would have been expressed, it would not have caused translation of the CFEM domain, which is the main focus of this study. Finally, qPCR analysis for the deleted gene showed that the selected strategy resulted in significant reduction of its expression by more than 90% in three independent deletion mutants (Figure 1d). Taken together, three independent ΔCFEM-*Bcin07g03260* mutants (from here on ΔCFEM-T4, ΔCFEM-T7 and ΔCFEM-T8) were used in downstream analyses.

### 2.7. Saprophytic Growth and Conidiation Assay

Saprophytic growth rates were measured on PDA Petri dishes (9 cm diameter) by inoculating them in the center with agar plugs of the tested *B. cinerea* isolates. Growth was marked twice a day between 3 to 5 days, before the culture reached the margins of the Petri dish. The average of two radius measurements per plate for each time point was used to calculate the growth area. Each isolate was tested in five replicates, and each experiment was conducted on three different dates (unless otherwise specified). To test conidiation, we inoculated (5 cm diameter) PDA plates with agar plugs containing different *B. cinerea* isolates, and incubated them for 10 days. Conidia were harvested by washing the cultured plates with sterile dH_2_O, filtering them through sterile cheesecloth, followed by precipitation (13,000 rpm, 1min), and resuspension in an equal volume (1 mL) of dH_2_O for all isolates. Each isolate (ΔCFEM deletion mutants, and WT) was tested in five replicates, and each experiment was conducted on three different dates. The average number of conidia of all dates was used to compare between isolates.

### 2.8. Conidial Germination Assay

For conidial germination, cultures (of ΔCFEM deletion mutants—T4 and T8, and WT isolates) were grown and conidia were later harvested as described above for the conidiation assay (Section 2.7). For the germination assay, conidia were adjusted to 1 × 10^5^ conidia/mL in ¼ PDB and incubated at 20 °C in the dark at 120 rpm in the presence or absence of 2.0 mg/L of the germination inhibitor pyrimethanil (causing EC_30_) (Mythos SC300, Bayer’s Crop Sciences, Germany). The rationale behind the low dosage of fungicide application was to implement a moderate stress on the conidial germination, which should enable the identification of a partial effect on germination in the mutant lines. Conidia’s germination was monitored at 3 and 6 hpi under a light microscope (Leica DMLB connected to Nikon DS fii camera). The experiment was repeated on three different dates, and each isolate was sampled five times. The length of the germ tube was calculated with ImageJ software (NIH).

### 2.9. Statistical Analysis

**Figure 2:**
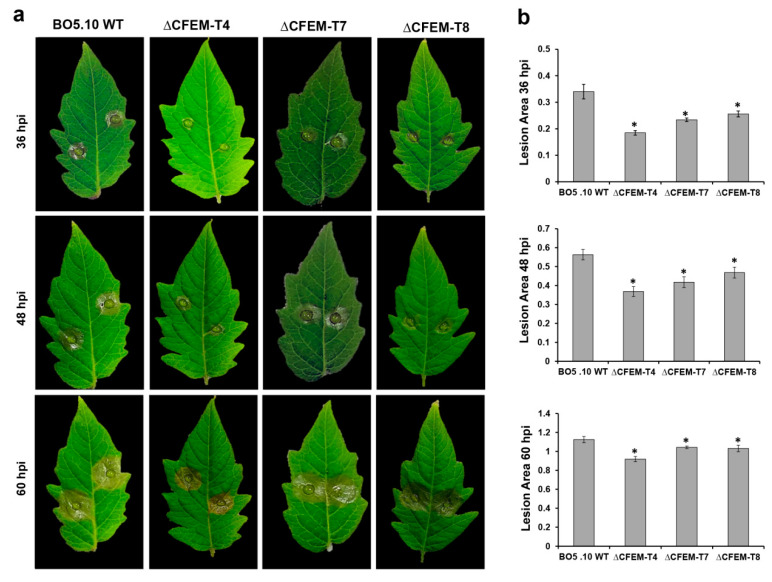
CFEM-Bcin07g03260 is required for pathogenicity in *B. cinerea*. (**a**) Deletion of the *CFEM-Bcin07g03260* gene caused reduced virulence on tomato leaves. Detached tomato leaves were inoculated with 10 µL drops of 10^5^ conidia/mL in ¼ PDB, and photographs were taken at 36, 48, and 60 hpi. (**b**) Average lesion area of the infected leaves with WT or ΔCFEM mutants at different time points. The experiment was repeated on three separate dates and the data was averaged. Each experiment was performed with *n* > 20. Error bars represent the ±SE of the mean. Asterisks indicate significant results between averages (*p* < 0.05, obtained by Student’s T-test).

**Figure 3:**
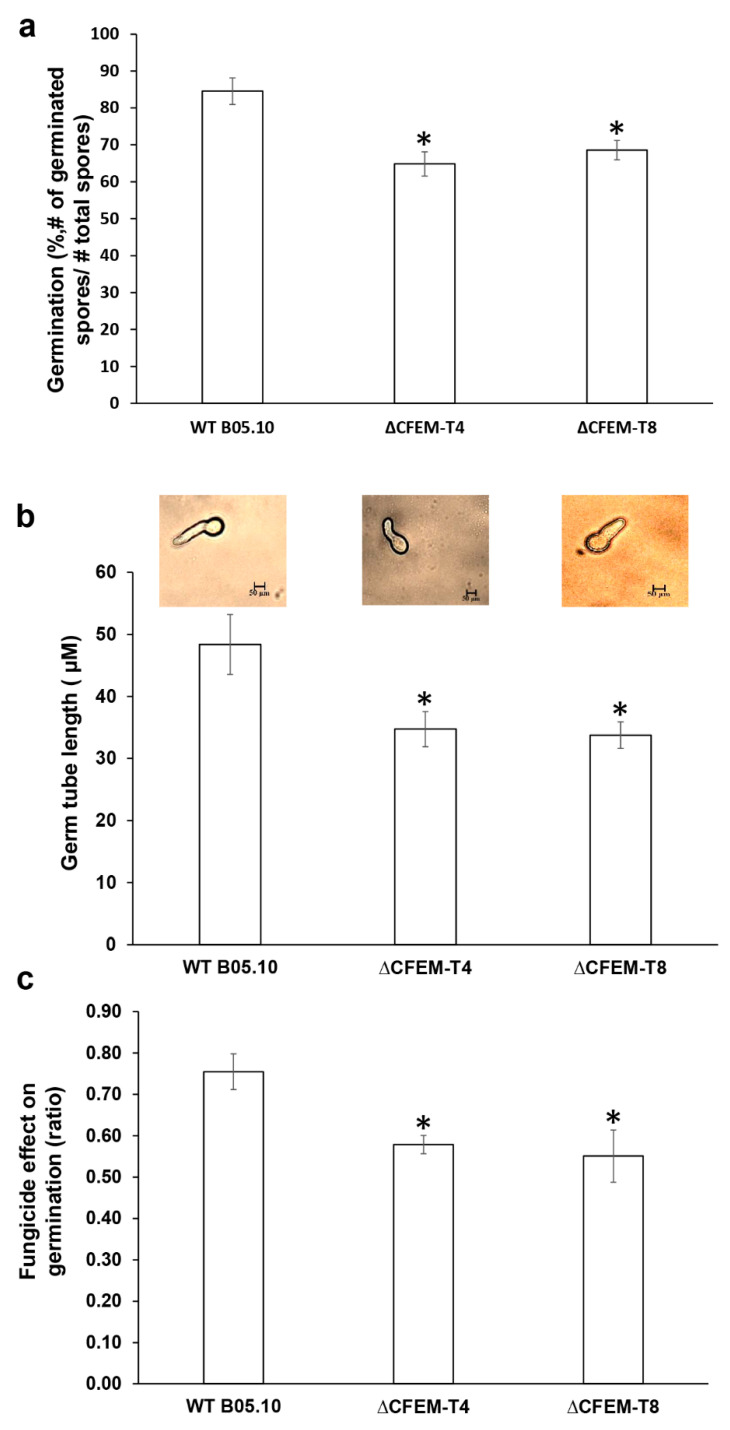
CFEM-*Bcin07g03260* is required for conidial germination and germ tube elongation. (**a**) Average conidial germination (%) in the WT, ΔCFEM-T4 and ΔCFEM-T8. Germination was monitored in ¼ PDB at 20 °C, 120 rpm in the dark. Germination (%) (Panel a) was calculated as (number of germinating conidia)/(total number of conidia). (**b**) Average germ tube length in the WT, ΔCFEM-T4, and ΔCFEM-T8 was calculated 6 h post inoculation in a similar condition to that described above. Photographs were taken at given time points, and germ tube length was measured using ImageJ software. (**c**) Conidial germination (%) in the presence of the fungicide pyrimethanil (2 mg/L). Conidial germination (panels a, and **c**) was monitored in the presence or absence of the fungicide. The experiment was repeated three times (*n* = 5 samples in each for conidial germination, and *n* = 50 for germ tube elongation). Error bars represent ±SE. Asterisks indicate significant difference between averages of the three different experiments (*p* < 0.05, obtained by Student’s T-test). To calculate the effect of fungicide on conidial germination, the ratio was calculated as (germination in the presence of fungicide)/(germination in the absence of fungicide).

**Figure 4:**
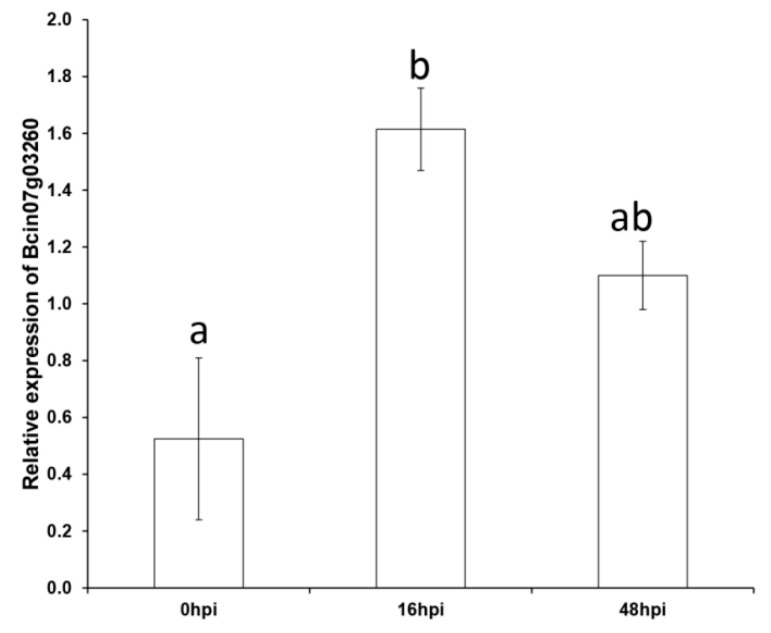
Expression levels of CFEM-*Bcin07g03260* encoding for the predicted membrane protein in WT. Gene expression levels were monitored by real-time qPCR from infected tomato leaf tissue of whole plants (at 0, 16, and 48 h post infection; two independent biological replicates per treatment). The y axis represents fold change compared with the housekeeping gene encoding for ubiquitin-conjugating enzyme (UCE), based on ΔCt values. ANOVA showed a significant difference between time points (*p* value < 0.05). Different letters indicate a significant difference between pairs (e.g., 0 hpi vs. 16 hpi) based on Tukey HSD (*p* value < 0.05). Error bars represent ±SE.

All experiments (unless otherwise specified) were carried out in triplicate and repeated three times. Average values and standard errors were calculated using Excel 2010 (Microsoft Inc., Seattle, WA, USA). When the question was whether each separate mutant was different from the WT strain (i.e., in results illustrated in Figure 1d, Figure 2, and Figure 3), the statistical analysis that was performed was the Student’s *T*-test. However, when the question of interest was whether more than two different treatments were significantly different from each other (i.e., in results illustrated in Figure 4 comparing expression in three infection times), we used the analysis of variance (one way ANOVA) followed by Tukey HSD. For all statistical tests, differences with *p* values < 0.05 were considered statistically significant (under the assumption of normality).

## 3. Results

### 3.1. Identification of Bcin07g03260, a Non-GPCR Membrane-Bound CFEM-Containing Protein

Our computational analysis identified three potential non-GPCR membrane bound CFEM-containing protein coding genes that contain the CFEM domain, including the conserved pattern of eight cysteines [10], the transmembrane domain(s), and a conserved aspartic residue [17,23] (Table 1, Materials and Methods). Among these three, Bcin07g03260 was predicted to contain only one transmembrane domain, while Bcin05g02420, and Bcin15g02580 proteins were predicted to contain 6 or 8 transmembrane domains, respectively (Table 1). Therefore, we selected the Bcin07g03260 homolog for further analyses, which has the highest dissimilarity to GPCR proteins that typically contain 7 transmembrane domains [24].

### 3.2. Study of ΔCFEM-Bcin07g03260 Mutants

To study the role of *Bcin07g03260* in *B. cinerea* (strain BO5.10) physiology and pathogenicity, we generated three independent deletion mutants of CFEM-*Bcin07g03260* via a homologous recombination based approach (ΔCFEM-T4, ΔCFEM-T7 and ΔCFEM-T8; see Materials and Methods, and Figure 1a–c). Results of real-time quantitative PCR confirmed that the expression of CFEM-*Bcin07g03260* in these mutants (ΔCFEM-T4, ΔCFEM-T7 and ΔCFEM-T8) is negligible (Figure 1d).

### 3.3. CFEM-Bcin07g03260 is not Required for Growth, and Conidiation of B. cinerea

To test the role of CFEM-Bcin07g03260 in hyphal growth of *B. cinerea*, we compared the growth area of WT and CFEM mutants on PDA plates (Table 3). No significant reduction was observed in growth of the two CFEM deletion mutants (ΔCFEM-T4, and ΔCFEM-T8) compared with the WT (tested at 24, 48, and 72 hpi). For example the average areas at 48 hpi were 15.71 ± 0.29, 15.55 ± 3.02, and 14.28 ± 3.14 cm^2^ for the WT, ΔCFEM-T4, and ΔCFEM-T8 strains respectively. Similarly, we did not identify significant differences in conidiation between the WT (42.8 × 10^6^ ± 5.18 conidia per plate), and the two CFEM deletion mutants (ΔCFEM-T4, 36.33 × 10^6^ ± 5.96; and ΔCFEM-T8, 39.38 × 10^6^ ± 7.74 conidia per plate) (Table 3).

### 3.4. CFEM-Bcin07g03260 is Required for Pathogenicity of B. cinerea

To study the effect of CFEM deletion on *B. cinerea* virulence, we compared the infection process of the different isolates on detached tomato leaves inoculated with conidia (Materials and Methods). During the first 24 hpi, we did not observe any visible symptoms in CFEM deletion mutants. However, clear visible infection regions were observed in the WT strain (Figure 2a). A significant reduction in the lesion size was observed in all three CFEM deletion mutants at 48 hpi (reduction in lesion area of 35% for ΔCFEM-T4, 25% for ΔCFEM-T7, and 18% in ΔCFEM-T8 compared with the WT BO5 isolate (Figure 2b).

### 3.5. CFEM-Bcin07g03260 is Required for Conidial Germination and Germ Tube Elongation

Since CFEM mutants exhibited reduced pathogenicity on tomato leaves, we were interested in studying the potential physiological mechanism involved in this phenotype. Therefore, we compared conidial germination of the WT with that of two deletion mutants. The CFEM mutants exhibited a significant reduction in conidia germination (23 and 19% reduction in ΔCFEM-T4 and ΔCFEM-T8, respectively) compared with the WT isolate (Figure 3a). In addition to the binary phenotype of germinating/non-germinating, we were also interested in studying potential difference in the germ tube elongation that proceeds the germination. The CFEM mutants exhibited a significant reduction in germ tube length (30 and 19% reduction in ΔCFEM-T4 and ΔCFEM-T8, respectively) compared with the WT (Figure 3b). To further study the role of germination in CFEM mutants, we monitored germination in the presence of the germination inhibitor pyrimethanil (2.0 mg/L). The CFEM mutants showed 70% more inhibition in germination than the WT (42 and 45% inhibition in ΔCFEM-T4 and ΔCFEM-T8, respectively, compared with 25% inhibition in the WT in the presence of the germination inhibitor pyrimethanil (Figure 3c)).

### 3.6. CFEM-Bcin07g03260 is Upregulated in the Course of Germination

To further study the involvement of the membrane CFEM encoding gene in germination, we monitored the expression of CFEM-*Bcin07g03260* in WT in early (16 hpi) and late stages (48 hpi) of the infection of tomato leaves, using qPCR with the gene specific primers. In line with the results showing the involvement of CFEM-*Bcin07g03260* in the conidial germination process, *Bcin07g03260* exhibited a three-fold increase in expression levels at the germination stage (16 hpi) compared with 0 hpi (Figure 4). This increase was followed by a decrease in expression level at the necrotic growth stage (48 hpi) (significant upregulation over 0 hpi was detected only by *T*-test but not by Tukey HSD test) (Figure 4).

## 4. Discussion

In the present study, we report the identification and functional characterization of a non-GPCR membrane bound CFEM-containing protein in *B. cinerea*. The gene coding for this protein is likely to be involved in plant pathogenicity, conidial germination, and consequent germ tube elongation.

Our computational analysis identified three potential non-GPCR membrane bound CFEM-containing protein coding genes, one of them contains a single transmembrane domain. To the best of our knowledge, this is the only single transmembrane CFEM-containing protein, and the only non-GPCR membrane CFEM-containing protein that has been analyzed in phytopathogenic fungi.

We bring several lines of evidence that illustrate that the non-GPCR membrane CFEM-containing protein, CFEM-Bcin07g03260, is likely to be involved in the virulence of *B. cinerea*. Three independent deletion mutants of CFEM-*Bcin07g03260* showed significantly reduced progression (above 20% reduction compared with WT) of the necrotic lesion during infection on tomato leaves. The CFEM deletion mutants exhibited a significant reduction (over 20%) in conidial germination (%) and in the following process of germ tube elongation compared with the WT isolate. Finally, we demonstrated that CFEM-*Bcin07g03260* gene expression is (up)regulated during the germination stage. The higher sensitivity of CFEM deletion mutants to the germination inhibitor further supports the role of the CFEM-*Bcin07g03260* gene in the germination stage. Together, these results demonstrate that an important part of the inhibition in virulence can be attributed to inhibition in germination and germ tube elongation. The authors acknowledge that future complementation of the *Bcin07g03260* gene that will show recovery of the phenotypes, could supply better proof that the phenotype in the KO (knock-out) mutant is not a result of a stochastic event such as integration of the KO construct into a non-targeted gene. However, in *B. cinerea*, random ectopic integration is considered a rare event that is technically challenging to achieve, while homologous recombination has a high frequency (see discussion in [8]). Thus, the homologous recombination construct used in this study to generate KO has a small chance for random ectopic integration, this probability is even lower in three independent mutants presented in this work.

Studies in phytopathogenic fungi have demonstrated the role of other membrane CFEM-containing proteins (CFEM GPCRs) in pathogenicity and physiology [18,25,26]. Deletion mutants of a GPCR CFEM-containing membrane protein (Pth11-like WISH protein) in *M. oryzae* were nonpathogenic due to a defect in sensing hydrophobic surface and appressorium differentiation [18]. The ΔFGRRES_16221 mutant of the CFEM-containing PTH11-like receptor in *F. graminearum* also showed reduced virulence, together with enhanced expression of the wheat host defensive response mechanism (e.g., chitinase) in the course of infection. Our current work in *B. cinerea*, complements previous study [19], which illustrated the requirement of secreted CFEM protein (named CFEM1) for pathogenicity of this pathogen. The Δ*CFEM1* mutant showed approximately 30% reduction in progression of necrotic lesions on bean leaves, and in production of conidia on PDA medium; however, germination was not affected [19]. These mutants showed increased sensitivity to salt (NaCl), osmotic (sorbitol) oxidative, and cell wall (SDS) stresses (H_2_O_2_) [19]. We tested conidia production, and sensitivity to these stresses in the membrane CFEM-*Bcin07g03260* deletion mutants used in our study under the same conditions [19]. In contrast with the secreted CFEM mutants (Δ*CFEM1* [19]), the membrane CFEM mutants tested in our work did not show sensitivity to these stresses nor reduced conidia production; however, it showed reduced germination and germ tube elongation. Thus, the two CFEM proteins are involved in different physiological pathways that are likely to complement each other to control the fungal virulence.

Germination of dormant conidia into hyphae is a crucial stage for successful completion of the disease cycle in air born fungal phytopathogens like *B. cinerea* [39]. Studies in *B. cinerea*, have demonstrated that this process can be induced by different combinations of nutrients and hydrophobic surfaces, and is regulated by components of the phosphorylative regulation mechanism [40,41,42,43]. In this study, both membrane CFEM mutants showed markedly reduced germination (compared with WT) at 3 hpi, and germ tube elongation at 6 hpi. Study of the kinetics of *B. cinerea* conidia germination has shown similar results, since the majority of conidia germinated at 2.5 h, and a majority of germ tube elongation was stopped after 4 h [44]. Differences between the timings of the stages may be a result of different conditions, i.e., rich liquid media used in this study compared with solid wax minimal fructose medium used in their study [44]. Interestingly, a CFEM-containing protein coding gene was shown to be upregulated in the course of germination (after 1 h) (see supplemental materials in [44]). This result was demonstrated in different conditions, i.e., germination on solid surface compared with liquid media in the current work, suggesting a central role of CFEM proteins functioning in both “pathways” of conidium germination.

Finally, recent network-based comparative genomics analysis of phytopathogenic fungi (and oomycetes) performed by our group has highlighted multiple functions involved in the pathogenicity of various pathogenic strategies [45]. If filtering for functions that were not present in the network is removed (third filter in Materials and Methods section, [45]), CFEM is significantly enriched in the core of the necrotrophic lifestyle (core comprises at least 70% of the necrotrophs). Together with our study, and previous works in necrotrophs, this result highlights the importance of CFEM proteins in the necrotrophic lifestyle.

## 5. Conclusions

CFEM proteins were shown to have various roles in (phyto)pathogenic fungi. In this study, we demonstrate the role of non-GPCR membrane bound CFEM-containing protein in necrotic growth, and germination of *B. cinerea*. One interesting future direction would be to explore the potential connection between known regulatory pathways of germination and the CFEM protein analyzed in this work. Other interesting direction would be to decipher the role of unstudied CFEM containing proteins in the virulence process of *B. cinerea* [45], and other phytopathogenic fungi.

## Figures and Tables

**Table 1:** Computational identification and characterization of CFEM-containing protein coding genes, which are associated with the membrane (Materials and Methods).

Gene ID (Ensembl)	TM ^1^	Sub-Cellularlocations ^2^	GPI Anchor Site Prediction [32]	Presence of Eight Cys Consensus Pattern	Contains Asp Aligned with Csa2′s Axial Asp	Final Location Prediction ^3^	Potential Non-GPCR
Bcin05g02420	6 TM domains	PM	No	Yes	Yes	MP	Medium
Bcin07g03260 ^s^	1 TM domain	PM	No	Yes	Yes	MP	Best
Bcin15g02580	7–8 TM domain ^4^	PM	No	Yes	Yes	MP	Worst

^1^ TM—transmembrane domain prediction [TMHMM, [31]. ^2^ Subcellular localization prediction using WoLF PSORT: PM—plasma membrane. ^3^ Final prediction abbreviations indicate: MP—membrane protein. ^4^ Containing only 7 transmembrane domains according to InterPro. ^s^ Selected for further research.

**Table 2:** Primers used in the study.

Name of Primer (FP, Forward Primer; RP, Reverse Primer)	Purpose	Sequence (5′→3′)
P131FP	5′ flank Integration	TACTGTGCAGTAGGTCGAGC
P126 RP	5′ flank Integration	CTTGCTTGACAAACGCACCA
P124FP	3′ flank Integration	CTCGGAGGGCGAAGAATCTC
P138RP	3′ flank Integration	GGGGAAGGTTTGGAAGGTGG
BcActin FP	Positive control for PCR	TGCTCCAGAAGCTTTGTTCCAA
BcActin RP	Positive control for PCR	TCGGAGATACCTGGGTACATAG
CFEM RT FP	CFEM qRT-PCR	AAGAGGAGGATGTGGGGTCA
CFEM RT RP	CFEM qRT-PCR	CTAGCACATCGACGTCCTCC
BcUBQ RT FP	Control for qRT-PCR	CAAGGTTACCGACAACAATA
BcUBQ RT RP	Control for qRT-PCR	GCATCCATCAACTTCTTCAA
BcUCE RT FP	Control for qRT-PCR	ATCACCCAAACATCAACT
BcUCE RT RP	Control for qRT-PCR	CATAGAGCAGATGGACAA
CFEM 5′Flank (BamHI) FP	To amplify 5′ Flank	CGCGGATCCCCGTGCTGGAGCATGTTGGCAC
CFEM 5′Flank (KpnI) RP	To amplify 5′ Flank	CGGGGTACCCCCCGGTCAGAATATGACATGG
CFEM 3′Flank (MscI) FP	To amplify 3′ Flank	TCGCGATGGCCAACACCTCGAACTCCAACCAC
CFEM 3′Flank (PstI) RP	To amplify 3′ Flank	AAAACTGCAGCAAACGCCACCATGATACAC

**Table 3:** Growth and sporulation in the BO5 WT and CFEM deletion mutants.

Isolate	Average Colony Area cm^2^ ± SE (*p* Value, Student’s T-test Against W)			Conidiation per Plate (i.e., per 19.6 cm^2^)
	24 hpi	48 hpi	72 hpi	14 dpi
BO5 WT	4.49 ± 1.54	15.72 ± 0.28	36.07 ± 0.29	42.8 ± 5.17
∆CFEM-T4	3.52 ± 1.58 (*p* ≤ 0.58)	15.54 ± 3.02 (*p* ≤ 0.94)	3 ± 0.25 (*p* ≤ 0.54)	36.39 ± 3.34 (*p* ≤ 0.45)
∆CFEM-T8	3.36 ± 1.90 (*p* ≤ 0.57)	14.28 ± 3.14 (*p* ≤ 0.57)	34.12 ± 3.82 (*p* ≤ 0.53)	39.37 ± 7.73 (*p* ≤ 0.73)
